# Acromegaly presenting with low insulin-like growth factor-1 levels and diabetes: a case report

**DOI:** 10.1186/s13256-015-0736-z

**Published:** 2015-10-30

**Authors:** Dilushi Rowena Wijayaratne, M. H. Arambewela, Chamara Dalugama, Dishni Wijesundera, Noel Somasundaram, Prasad Katulanda

**Affiliations:** 1 University Medical Unit, National Hospital of Sri Lanka, Colombo, Sri Lanka; 2 Diabetes and Endocrine Unit, National Hospital Sri Lanka, Colombo, Sri Lanka

**Keywords:** Acromegaly, Diabetes, Insulin-like growth factor-1

## Abstract

**Introduction:**

Acromegaly is an endocrine disorder arising from excessive serum growth hormone levels in adulthood and is characterized by progressive somatic enlargement. Biochemical confirmation is achieved by demonstration of elevated baseline serum growth hormone levels which are not suppressed during an oral glucose tolerance test, and by increased levels of serum insulin-like growth factor-1. The serum insulin-like growth factor-1 level provides an assessment of integrated growth hormone secretion and is recommended for diagnosis, monitoring, and screening of acromegaly. We report a case of a patient with acromegaly secondary to a pituitary microadenoma who presented with low insulin-like growth factor-1.

**Case presentation:**

An 83-year-old Sinhalese woman presented to our hospital with an enlarging multinodular goiter. She was observed to have macroglossia, thickened coarse skin, acral enlargement, and newly detected, uncontrolled diabetes. A diagnosis of acromegaly was suspected. She did not complain of recent headaches, vomiting, visual difficulties, or galactorrhea and was clinically euthyroid. Her pulse rate was 84 beats/min, and her blood pressure was 150/90 mmHg. A visual field assessment did not reveal a defect. Her random growth hormone levels were 149 mU/L (<10 mU/L), and her oral glucose tolerance test was supportive of acromegaly with a paradoxical rise of growth hormone. Her serum age-specific insulin-like growth factor-1 level was below normal at 124.7 ng/ml (normal range 150–350 ng/ml). Her serum insulin-like growth factor-1 level, measured after glycemic control was achieved with metformin and insulin, was elevated, which is characteristic of acromegaly. Magnetic resonance imaging of her pituitary revealed a pituitary microadenoma. Acromegaly secondary to a growth hormone–secreting pituitary microadenoma was confirmed.

**Conclusions:**

Systemic illnesses, including catabolic states, hepatic or renal failure, malnutrition, and diabetes mellitus, are known to decrease insulin-like growth factor-1 levels and may result in false-negative values in patients with acromegaly A low insulin-like growth factor-1 level does not exclude acromegaly in a patient with supportive clinical features and poorly controlled diabetes.

## Introduction

Acromegaly is an endocrine disorder characterized by progressive somatic disfigurement involving mainly the face and extremities. It arises from an abnormal elevation in serum growth hormone (GH) in adulthood and is often due to a pituitary adenoma [1]. Many of the actions of GH on somatic growth and tissue maintenance are mediated by insulin-like growth factor-1 (IGF-1), which is produced by the liver in response to GH [2]. The diagnosis is confirmed biochemically by the presence of elevated serum GH levels that are not suppressed after an oral glucose tolerance test (OGTT), as well as by detection of increased IGF-1 levels [1]. The serum IGF-1 level is a good tool to use for assessment of integrated GH secretion and is recommended for diagnosis, monitoring, and screening of acromegaly [3]. Systemic illnesses, including catabolic states, hepatic or renal failure, malnutrition, and diabetes mellitus, may decrease the IGF-1 level and result in false-negative values in screening for acromegaly [3]. We report a case of a patient with acromegaly who presented with elevated GH, low IGF-1, and poorly controlled diabetes.

## Case presentation

An 83-year-old Sinhalese woman presented to our hospital for evaluation of a progressively enlarging, multinodular goiter. She was clinically euthyroid and reported no recent change in weight, cold or heat intolerance, alteration of bowel habits, fatigue, or mood changes. She was receiving treatment for hypertension with hydrochlorothiazide.

Her physical examination demonstrated that she was mildly dehydrated and her body mass index was 24 kg/m^2^. It also revealed a multinodular goiter with evidence of retrosternal extension and no cervical lymphadenopathy. Her pulse was regular at 84 beats/min, and her blood pressure was 150/90 mmHg. We observed her to have macroglossia, coarse thickened skin, and large, spade-like hands and feet. These findings raised the suspicion of acromegaly. However, she had not noticed a recent change in appearance. She did not describe having had recent headaches, vomiting, visual difficulties, or galactorrhea to suggest a pituitary neoplasm. She was blind in her right eye due to chronic glaucoma, but she had no obvious visual field defect upon examination.

Radiological and histological evaluations of her thyroid revealed a benign, multinodular goiter with early retrosternal extension. Her serum third-generation thyroid-stimulating hormone level was 0.017 μIU/ml (reference range 0.4–4.0 μIU/ml), her serum free thyroxine level was 1.73 ng/dl (reference range 0.89–1.76 ng/dl), and her free triiodothyronine level was 3.12 pg/ml (reference range 1.50–4.10 pg/ml). She was commenced on a daily carbimazole dose of 15 mg, as treatment for thyrotoxicosis. Basic investigations revealed that her hemoglobin level was 12.4 g/dl, her serum creatinine level was 108 μmol/L, and her electrolytes and liver function were normal. Her fasting blood glucose was 43 mmol/L, and she had no evidence osmotic symptoms. She had had a normal fasting blood glucose level when last screened 2 years before. Her urine test was negative for ketone bodies. At the outset, she required 80–90 U/day of insulin for glycemic control. Later, she was stabilized on 40 U/day of insulin together with 2 g/day of metformin.

On the basis of her physical features, together with newly detected diabetes, a diagnosis of acromegaly was suspected. However, screening for serum IGF-1 using a commercially available enzyme-linked immunosorbent assay revealed that her level was low at 124.7 ng/ml (normal range for adults 150–350 ng/ml as specified on product leaflet, assay sensitivity 1.29 ng/ml, intra-assay coefficient of variation 6.62 %, inter-assay coefficient of variation 7.79 %, sample preparation by incubation in 1 M hydrochloric acid).

Her random GH levels were elevated at 149 mU/L (normal range<10 mU/L), and her OGTT demonstrated a paradoxical rise of GH (Table 1). Though the OGTT is known to yield false-positive results in patients with diabetes, the very high baseline levels of GH in our patient were suggestive of acromegaly. In this setting, the low serum IGF-1 level could be attributed to poorly controlled diabetes.

**Table 1:** Results of oral glucose tolerance test performed for diagnosis of acromegaly after 15 days of insulin therapy

Time from administration of 75 g anhydrous glucose	Growth hormone (mU/L)	Venous glucose (mmol/L)
0 hours	88.1	9
1 hours	>100	13.4
2 hours	98.9	17.8

IGF-1 measurements were repeated after glycemic control was achieved with insulin and metformin. Her IGF-1 level had risen above the normal range (Table 2), confirming acromegaly beyond doubt.

**Table 2:** Variation of serum insulin-like growth factor-1 with glycemic control

Days since initiation of insulin	Serum insulin-like growth factor-1^a^ (ng/ml)	Fasting venous blood glucose (mmol/L)
Day 4	124.7	26
Day 22	265.10	9
Day 45	504	5.3

^a^Reference range for adults 150–350 ng/ml

Magnetic resonance imaging of the patient’s pituitary revealed an 8-mm pituitary microadenoma. Visual perimetry was confounded by a cataract in one eye and chronic glaucoma in the other. Her pituitary hormone axes, other than the thyroid axis, were normal.

The patient was diagnosed with acromegaly secondary to a GH-secreting pituitary microadenoma associated with a toxic multinodular goiter. She was commenced on treatment with cabergoline 500 μg/wk, as she was unwilling to undergo pituitary surgery and radiotherapy and because somatostatin analogues and pegvisomant were not routinely available at our hospital.

## Discussion

Apart from its somatic effect, GH has important effects on body metabolism, including glucose homeostasis [4]. GH acts at several levels, including the insulin receptor and its postreceptor signal transduction pathways, to block insulin actions, leading to reduced insulin-driven glucose uptake in peripheral tissue and suppression of gluconeogenesis. Increased lipolysis in the presence of GH reduces glucose oxidation by acting as a competitive energy source [5]. However, whereas these mechanisms contribute to hyperglycemia, IGF-1, its peripheral target hormone, has opposing effects. Excess GH causes insulin resistance and hyperglycemia, whereas IGF-1 has insulin-like effects that reduce blood glucose levels. In fact, IGF-1 has been used experimentally to treat hyperglycemia of both type 1 and type 2 diabetes [6]. Thus, in acromegaly, the final level of glycemia is dependent on the balance between the activities of GH and IGF-1.

There is evidence to suggest that insulin enhances IGF-1 production by either direct regulation of the GH receptor or a permissive effect on postreceptor events [7]. In keeping with this finding, normal IGF-1 levels have been reported in patients with poorly controlled diabetes who present with acromegaly [8], and it is recommended that IGF-1 be measured after hyperglycemia is controlled [3].

Our patient had a low IGF-1 level upon presentation, and her diagnosis could have been missed if not for her grossly elevated baseline GH.

Considering that IGF-1 is known to be responsible for most of the tissue trophic effects of GH, it is counterintuitive that patients with low IGF-1 can present with features of acromegaly. It is possible that a direct action of GH on peripheral tissue growth could account for our patient’s physical features, regardless of her IGF-1 levels. The relative or absolute insulin deficiency of diabetes may have rendered her less sensitive to the insulin-enhanced hepatic production of IGF-1 and may explain why her IGF-1 levels increased with insulin and metformin treatment. This may have been preceded by a period in which the balance between IGF-1 and GH favored the development of acromegalic features.

Low IGF-1 in the presence of clinical acromegaly could also represent a later stage of a disease process that was initially associated with elevated IGF-1, resulting in clinical features of acromegaly, but that has now “burnt out” (that is, burnt-out acromegaly). In our patient, the rise in IGF-1 after glycemic control was achieved contradicted this possibility. Further, burnt-out acromegaly is often accompanied by other features of hypopituitarism, which were absent in our patient.

## Conclusions

We present a case of an elderly patient with acromegaly and poorly controlled diabetes with low IGF-1 at presentation. A low IGF-1 level does not exclude acromegaly in a patient with supportive clinical features and poorly controlled diabetes. Patients with diabetes may present with only mild features of acromegaly, which may be disregarded as being due to aging or diabetic pseudoacromegaly. A high index of suspicion must be maintained to avoid missing this important diagnosis.

## Consent

Written informed consent was obtained from the patient for publication of this case report and any accompanying images. A copy of the written consent is available for review by the Editor-in-Chief of this journal.

## Abbreviations

GH: Growth hormone
IGF-1: Insulin-like growth factor-1
OGTT: Oral glucose tolerance test
